# Serum Taurine and Stroke Risk in Women: A Prospective, Nested Case-Control Study

**DOI:** 10.1371/journal.pone.0149348

**Published:** 2016-02-11

**Authors:** Fen Wu, Karen L. Koenig, Anne Zeleniuch-Jacquotte, Saran Jonas, Yelena Afanasyeva, Oktawia P. Wójcik, Max Costa, Yu Chen

**Affiliations:** 1 Department of Population Health, New York University School of Medicine, 650 First Avenue, New York, New York, United States of America; 2 Department of Neurology, New York University School of Medicine, 462 First Avenue, New York, New York, United States of America; 3 Robert Wood Johnson Foundation, Route 1 and College Road East, Princeton, New Jersey, United States of America; 4 Department of Environmental Medicine, New York University School of Medicine, 57 Old Forge Rd, Tuxedo Park, New York, United States of America; National Cancer Center, JAPAN

## Abstract

**Background:**

Taurine (2-aminoethanesulfonic acid), a conditionally essential sulfur-containing amino acid, is mainly obtained from diet in humans. Experimental studies have shown that taurine’s main biological actions include bile salt conjugation, blood pressure regulation, anti-oxidation, and anti-inflammation.

**Methods:**

We conducted a prospective case-control study nested in the New York University Women’s Health Study, a cohort study involving 14,274 women enrolled since 1985. Taurine was measured in pre-diagnostic serum samples of 241 stroke cases and 479 matched controls.

**Results:**

There was no statistically significant association between serum taurine and stroke risk in the overall study population. The adjusted ORs for stroke were 1.0 (reference), 0.87 (95% CI, 0.59–1.28), and 1.03 (95% CI, 0.69–1.54) in increasing tertiles of taurine (64.3–126.6, 126.7–152.9, and 153.0–308.5 nmol/mL, respectively). A significant inverse association between serum taurine and stroke risk was observed among never smokers, with an adjusted OR of 0.66 (95% CI, 0.37–1.18) and 0.50 (95% CI, 0.26–0.94) for the second and third tertile, respectively (*p* for trend = 0.01), but not among past or current smokers (*p* for interaction < 0.01).

**Conclusions:**

We observed no overall association between serum taurine and stroke risk, although a protective effect was observed in never smokers, which requires further investigation.

Taurine, Stroke, Epidemiology, Prospective, Case-control study, NYUWHS.

## Introduction

Stroke is one of the leading causes of death and disability in the world. Large prospective epidemiologic studies have provided evidence that nutritional factors such as antioxidants and healthy dietary patterns are important modifiable risk factors for stroke [1,2]. Identification of a novel nutrient from diet that can protect against stroke risk may improve stroke prevention and enhance our understanding of its pathophysiology.

Taurine (2-aminoethanesulfonic acid) is a conditionally essential sulfur-containing amino acid mostly obtained from diet, predominantly seafood and the dark meat of poultry [3]. Experimental studies have suggested that taurine is involved in bile salt conjugation, blood pressure regulation, anti-oxidation, and anti-inflammation [4]. Several clinical trials have indicated that taurine may lower cholesterol level and blood pressure [5–7]; however, these trials were limited by small sample sizes, short-term duration of follow-up, and selection of participants with existing cardiovascular risk factors such as obesity and hypertension. A few cross-sectional studies have reported an inverse ecologic correlation of group means of urinary excretion of taurine, a surrogate of taurine intake, with body mass index, blood pressure, cholesterol levels, and mortality rate due to stroke [8–11]. However, the observed correlations could be the result of ecologic fallacy because factors associated with disease rates at the group level may not be associated with disease at the individual level. In addition, the correlations may be confounded by unmeasured factors related to both taurine intake and stroke at the individual level.

We conducted a prospective case-control study nested in the New York University Women’s Health Study (NYUWHS) to examine the association between pre-diagnostic serum levels of taurine and subsequent stroke risk.

## Methods

### Study population

Details of the NYUWHS have been presented elsewhere [12]. Briefly, a total of 14,274 women, aged 34–65 years, were enrolled between 1985 and 1991 at a breast cancer screening center in New York City. Demographic, lifestyle, dietary, and medical information was collected using a self-administered questionnaire. Active follow-up of the cohort is conducted with questionnaires mailed every 2–4 years. The response rate for the last round of follow-up was 83% of the original cohort members.

Physical activity was assessed at baseline, and in two follow-up questionnaires in which participants were directed to report their activity levels during the baseline period. Data were coded to reflect the metabolic equivalent task (MET) hours per week of vigorous exercise, walking, housework, and occupational activity. Prevalent cases of hypertension and diabetes at baseline were identified using self-report information on physician-diagnosis of the condition, and/or medicine use for these conditions.

Non-fasting blood was collected at enrollment and stored at -80°C. Yearly blood samples are available for > 50% of the participants (*n* = 7,344). Written informed consent was obtained from all subjects. The study procedures were approved by the IRB of New York University School of Medicine.

### Selection of cases and controls

During follow-up, participants who reported having had carotid endarterectomy (CEA) or stroke were contacted for further information and authorization to obtain their medical records, which were reviewed by a neurologist and a cardiologist. Stroke cases included women who had documented non-fatal or fatal stroke, or CEA (whichever was earlier). Fatal stroke was confirmed if the underlying cause of death from the National Death Index (NDI) was cerebrovascular disease coded according to the International Classification of Diseases, 9th revision (ICD-9; codes 430–438) or 10th revision (ICD-10; codes I60–69.9). Non-fatal stroke was confirmed if it met the World Health Organization criteria of “rapidly developing clinical signs of focal disturbance of cerebral function, lasting more than 24 hours with no apparent cause other than that of vascular origin”. Strokes were classified as ischemic, hemorrhagic, or stroke of undetermined type, based on information in the medical charts.

We identified 241 incident cases of stroke who were free of CVD at baseline. For each stroke case, two controls were selected at random from the risk set of NYUWHS subjects who were free of CVD at baseline and free of stroke as of the date of the stroke event of the case, and who matched the case on age (± 6 months) at baseline, menopausal status (pre- or post-menopausal), number of blood donations (1 or 2+), and dates (± 6 months) of blood donations.

### Measurement of serum taurine

Taurine was determined using high performance liquid chromatography (HPLC) (Waters, Millford, MA) with precolumn derivitization using o-phthaldialdehyde (Sigma-Aldrich, St. Louis, MO) and 3-mercaptopropionic acid (Sigma-Aldrich, St. Louis, MO) and fluorescence detection, as previously described [13]. The coefficient of variation (CV) of the assay, computed from the measurement of duplicate aliquot pairs, was 7% [14]. As mentioned, yearly blood samples are available for > 50% of the participants. To improve the long-term reproducibility of serum taurine, we measured taurine in two yearly serum samples for the case-control sets with two or more serum donations (140 cases and 277 controls) [15,16].

### Statistical analysis

We first compared the distribution of baseline characteristics and established risk factors for CVD by case-control status using conditional logistic regression model which takes into account the matching factors [17]. We used conditional logistic regression to estimate odds ratios (ORs) and their 95% confidence intervals (CIs) for stroke in relation to tertiles of serum taurine. We also estimated the ORs in association with a 1-SD increase in serum taurine for better interpretation of the effect estimates. For subjects with two yearly samples, we used the average of serum taurine from the two samples.

In addition to crude models (model 1), ORs were adjusted for body mass index (BMI) and smoking status (model 2), which are major risk factors for stroke. In a previous study, we found an inverse association of taurine with hypertension and diabetes [16]. Therefore, we conducted sensitivity analyses controlling for these conditions (data not shown) as well as excluding women with prevalent hypertension (model 3) or diabetes (model 4) at baseline. Sensitivity analyses were also conducted excluding stroke cases diagnosed within three years since baseline (model 5), under the assumption that individuals who are seriously ill at baseline may be more likely to have stroke in the first three years of follow-up. These analyses were conducted using unconditional logistic regression to maximize the power. Tests for linear trend were conducted using an ordered variable for tertile categories of serum taurine.

We conducted stratified analyses to examine whether the association between serum taurine and stroke differed by baseline age, BMI, or smoking status (never, past, and current). Age and BMI were dichotomized by the median value in the overall population. Multiplicative interaction was tested using a cross-product term between a potential effect modifier and serum taurine. ORs were also estimated for ischemic and hemorrhagic stroke separately, using unconditional logistic regression with adjustment for baseline age, menopausal status, BMI, and smoking status. All analyses were completed using SAS (version 9.3; SAS Institute Inc, Cary, NC).

## Results

Serum samples were stored for an average of 26 years. At the time of baseline blood donation, the median age of the study participants was 59.2 years, and stroke events occurred at an average age of 72.6 years. Compared with controls, women who subsequently developed stroke had a significantly higher BMI, and were more likely to be current smokers and to have hypertension and diabetes at baseline (*p* values < 0.05) (Table 1). The distribution of cases and controls did not differ by tertile categories of serum taurine (*p* = 0.58). Among the controls, mean serum taurine adjusted for baseline age differed significantly by smoking status (*p* < 0.05), with both past and current smokers having significantly lower levels of serum taurine than never smokers (data not shown).

**Table 1:** Baseline characteristics of stroke cases and controls.

Baseline characteristics	Cases (*n* = 241)^a^	Controls (*n* = 479)^a^	*P* value^b^
Age at baseline (years)	59.2 (48.0–64.3)	59.2 (47.7–64.4)	Matched
Menopausal status at enrollment			
Premenopausal	44 (18.3)	88 (18.4)	Matched
Postmenopausal	197 (81.7)	391 (81.6)	
Length of sample storage (years)	26.3 (24.6–27.2)	26.3 (24.7–27.0)	Matched
BMI (kg/m^2^)	25.1 (21.2–32.3)	24.7 (20.5–31.3)	0.05
Race			
Caucasian	172 (79.6)	349 (80.6)	0.26
Other	44 (20.4)	84 (19.4)	
Education			
≤ High school	98 (47.3)	158 (39.1)	0.23^c^
Some college or equivalent	44 (21.3)	102 (25.3)	
College degree and higher	65 (31.4)	144 (35.6)	
Physical activity (MET-hours/week)	8.6 (0.44–49.2)	11.0 (0.77–56.1)	0.41
Smoking status			
Never	94 (41.6)	222 (49.1)	< 0.01^c^
Past	76 (33.6)	171 (37.8)	
Current	56 (24.8)	59 (13.1)	
Prevalent hypertension			
No	136 (62.7)	334 (74.5)	< 0.01
Yes	81 (37.3)	114 (25.5)	
Prevalent diabetes			
No	203 (89.4)	447 (95.7)	< 0.01
Yes	24 (10.6)	20 (4.3)	
Serum taurine (nmol/mL)			
64.3–126.6	84 (34.9)	156 (32.6)	0.58
126.7–152.9	74 (30.7)	166 (34.7)	
153.0–308.5	83 (34.4)	157 (32.8)	

^a^Values are *n* (%) or median (10th and 90th percentiles) where indicated.

^b^*P* values were obtained from logistic regression conditional on the matching factors including age, menopausal status, number and dates of blood donations excluding those with missing information.

^c^Variables with more than two categories were entered as ordered categorical.

Overall, there was no association between serum taurine and stroke risk (Table 2). The adjusted ORs for stroke associated with increasing tertiles of serum taurine were 1.0 (reference), 0.87 (95% CI, 0.59–1.28), and 1.03 (95% CI, 0.69–1.54), respectively (model 2; *p* for trend = 0.89). The adjusted OR for stroke in association with a 1-SD increase in taurine (33 nmol/mL) was 1.03 (95% CI, 0.88–1.20; data not shown). Additional control for prevalent hypertension and diabetes at baseline, as well as daily consumption of major food groups (meat, poultry, fish, fruits, vegetables, cereals, and dairy products) did not materially change the results (data not shown). The associations remained similar after excluding women with hypertension (*n* = 195; models 3) or diabetes (*n* = 44; model 4) at baseline, or those diagnosed with stroke within three years of follow-up (*n* = 35; model 5).

**Table 2:** Association [OR (95% CI)] between mean taurine and stroke risk.

	Taurine tertile 1^a^	Taurine tertile 2^a^	Taurine tertile 3^a^	*p* for trend
Overall				
Case/control	84/156	74/166	83/157	
Model 1^b^	1.00	0.84 (0.58–1.23)	1.01 (0.68–1.50)	1.00
Model 2^c^	1.00	0.87 (0.59–1.28)	1.03 (0.69–1.54)	0.89
Model 3^d^	1.00	0.78 (0.47–1.30)	1.09 (0.67–1.77)	0.72
Model 4^e^	1.00	0.85 (0.56–1.29)	1.04 (0.69–1.57)	0.83
Model 5^f^	1.00	0.88 (0.59–1.30)	1.06 (0.72–1.57)	0.76
Ischemic stroke				
Case/control	29/156	24/166	30/157	
Model 1^g^	1.00	0.78 (0.44–1.40)	1.05 (0.60–1.84)	0.87
Model 2^h^	1.00	0.77 (0.43–1.38)	1.09 (0.62–1.92)	0.77
Hemorrhagic stroke				
Case/control	20/156	17/166	14/157	
Model 1^g^	1.00	0.78 (0.40–1.55)	0.67 (0.32–1.37)	0.26
Model 2^h^	1.00	0.81 (0.41–1.62)	0.71 (0.34–1.48)	0.36

^a^Taurine tertile 1: 64.3–126.6 nmol/mL; tertile 2: 126.7–152.9 nmol/mL; tertile 3: 153.0–308.5 nmol/mL.

^b^ORs were calculated using unadjusted logistic regression conditional on matching factors including age, menopausal status, and number and dates of blood donations.

^c^ORs were calculated using logistic regression conditional on matching factors with additional adjustment for BMI and smoking.

^d^ORs were calculated using unconditional logistic regression with adjustment for age, menopausal status, BMI, and smoking excluding women with hypertension at baseline (*n* = 195).

^e^ORs were calculated using unconditional logistic regression with adjustment for age, menopausal status, BMI, and smoking excluding women with diabetes at baseline (*n* = 44).

^f^ORs were calculated using unconditional logistic regression with adjustment for age, menopausal status, BMI, and smoking excluding women who developed stroke within three years since baseline (*n* = 35).

^g^ORs were calculated using unconditional logistic regression with adjustment for age and menopausal status.

^h^ORs were calculated using unconditional logistic regression with adjustment for age, menopausal status, BMI, and smoking.

The pattern of the associations between serum taurine and ischemic stroke (*n* = 83) was generally similar as that in the overall analysis (Table 2). For instance, the adjusted ORs in relation to increasing tertiles of serum taurine were 1.0 (reference), 0.77 (95% CI, 0.43–1.38), and 1.09 (95% CI, 0.62–1.92), respectively (model 2; *p* for trend = 0.77). On the other hand, serum taurine was non-significantly inversely related to hemorrhagic stroke (*n* = 51), with a 1-SD increase in serum taurine corresponding to an OR of 0.89 (95% CI, 0.66–1.21; data not shown); the adjusted ORs associated with increasing tertiles of serum taurine were 1.0 (reference), 0.81 (95% CI, 0.41–1.62), and 0.71 (95% CI, 0.34–1.48), respectively (model 2; *p* for trend = 0.36).

**Fig 1:**
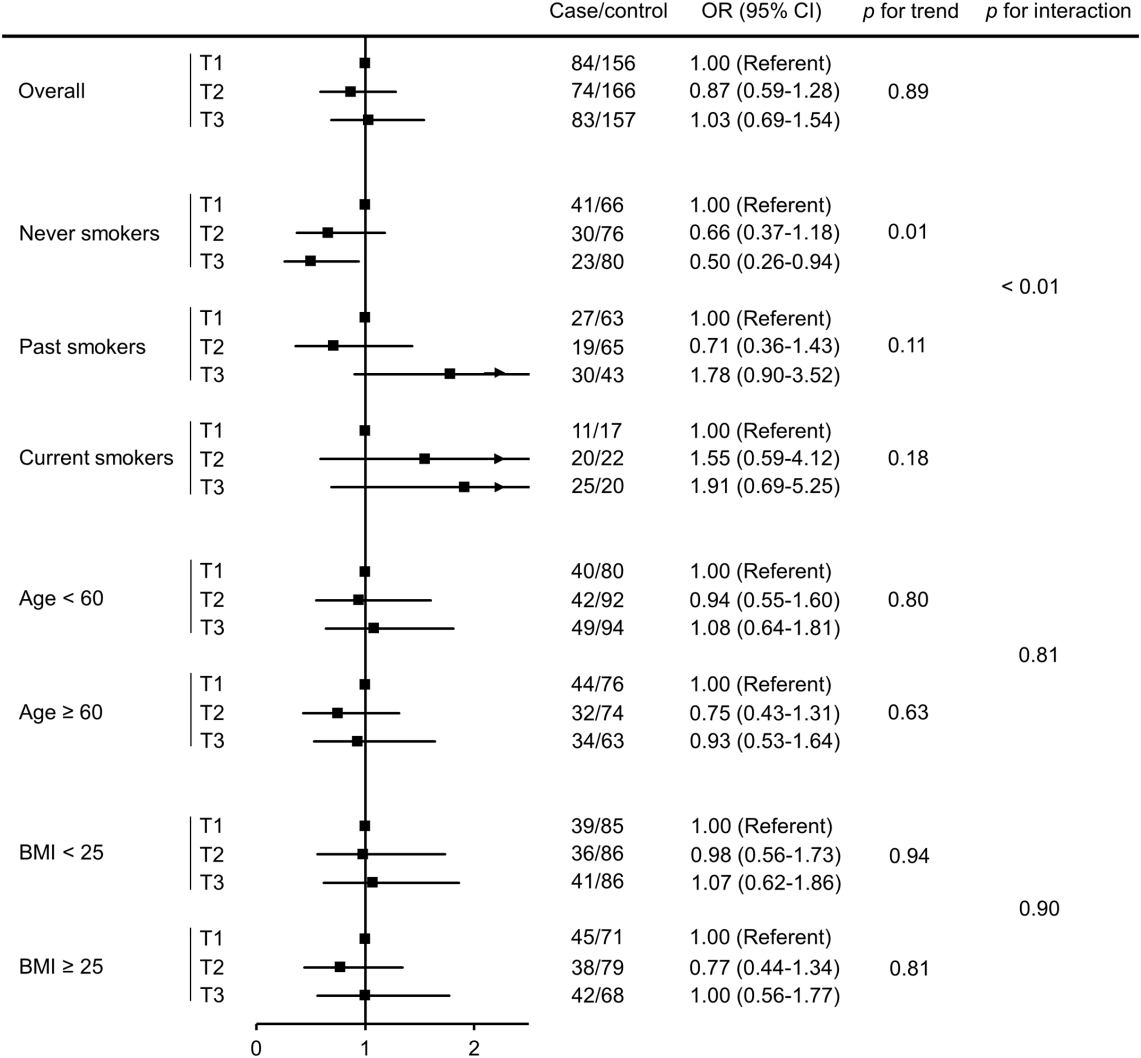
Association between mean taurine and stroke risk overall and stratified. Cut-off points for taurine were determined by tertiles (T1: 64.3–126.6 nmol/mL; T2: 126.7–152.9 nmol/mL; T3: 153.0–308.5 nmol/mL) in the overall population. ORs were calculated using logistic regression conditional on matching factors including age, menopausal status, number and dates of blood donations with additional adjustment for BMI and smoking (except for variables stratified by in stratified analyses). The arrows (for past and current smokers) indicate that the upper limit of the 95% confidence interval was not shown in the figure.

The association between serum taurine and stroke risk did not differ appreciably according to baseline age (*p* for interaction = 0.81) or BMI levels (*p* for interaction = 0.90) (Fig 1). Among never smokers, serum taurine was significantly inversely related to stroke risk (*p* for trend = 0.01). Compared with women whose taurine level was in the bottom tertile, those in the highest two tertiles of serum taurine were less likely to develop stroke, with an OR of 0.66 (95% CI, 0.37–1.18) and 0.50 (95% CI, 0.26–0.94) for the second and third tertile, respectively. On the other hand, there was no association between serum taurine and stroke risk in past or current smokers, with wide confidence intervals for the effect estimates. The interaction between taurine and smoking in stroke risk was significant (*p* for interaction < 0.01). Stratified analyses by smoking intensity (never, light, and heavy) produced similar effect estimates as those based on smoking status and therefore the results are not shown.

## Discussion

To the best of our knowledge, this is the first prospective study to examine the relationship between serum taurine and stroke risk. Overall, there was no association between serum taurine and stroke risk. However, we found a significant inverse association among never smokers, which was not observed in past or current smokers.

In the WHO Cardiovascular Disease and Alimentary Comparison (CARDIAC) Study with random samples from 61 populations in 25 countries, there was a non-significant correlation between average urinary taurine and stroke mortality at the group level in men [18]; however, further detailed analyses using structural equation modeling revealed a significant inverse association between the group means of 24-hour urinary taurine to creatinine ratio and stroke mortality in both men and women [8]. It should be noted that the CARDIAC study included a substantial number of populations from Asia, where the incidence of hemorrhagic stroke is much greater comparing with Western populations [19,20]. In the present study, we observed a non-significant inverse association between serum taurine and the risk of hemorrhagic stroke; however the sample size for subtype analyses was limited. Future large studies of stroke subtypes are needed given that stroke is a heterogeneous disease that comprises subtypes with different etiologies.

In subgroup analyses, we found that taurine may be protective against stroke risk in women who have never smoked. In the previous study on serum taurine and coronary heart disease (CHD) risk, we did not find an overall association, although the association was stronger among never smokers, with an OR of 0.78 (95% CI, 0.39–1.57) and 0.52 (95% CI, 0.24–1.09) for CHD comparing the higher two tertiles of taurine to the bottom tertile, respectively [16]. We also evaluated the association between taurine and combined endpoint CVD, defined as having either CHD or stroke, using data from both studies. A similar pattern of ORs was observed. For instance, the adjusted ORs for CVD associated with increasing tertiles of serum taurine were 1.0 (reference), 0.86 (95% CI, 0.64–1.17), and 1.00 (95% CI, 0.72–1.39), respectively. Among never smokers, serum taurine was significantly inversely related to CVD risk (*p* for trend < 0.01). These data suggest that in our study population, taurine was not related to stroke, CHD, or CVD in general, but the beneficial role may be more evident in never smokers. Previous cross-sectional and ecologic data on the inverse association between taurine and CHD was more consistent [8,18,21,22] than that between taurine and stroke [8,18]. However, there is no evidence that taurine may be more protective against specific CVD subtype. Future prospective studies investigating different subtypes of CVD are needed.

In our study, having had diabetes was significantly positively associated with subsequent stroke risk in the overall study population and within each smoking status. Serum taurine was inversely related to diabetes status in overall and ever smokers, but not in never smokers (data not shown). When we further adjusted for diabetes status in the stratified analyses of taurine and stroke risk by smoking status, the results do not materially change. For instance, among never smokers, serum taurine remained significantly inversely related to stroke risk, with an OR of 0.64 (95% CI, 0.35–1.18) and 0.50 (95% CI, 0.26–0.96) comparing the higher two tertiles of taurine to the bottom tertile (*p* for trend < 0.01), respectively. The data did not suggest that the association between serum taurine and stroke risk among never smokers was due to the effects of taurine on diabetes, although our data may be limited in assessing epidemiologic medication.

The potential beneficial effects of taurine on CVD risk are supported by its cholesterol-lowering, anti-hypertensive, anti-inflammatory and anti-oxidative actions demonstrated in many experimental and human studies. Specifically, taurine prevents hyperlipidemia by conjugation with bile acids and enhancement of bile acid production by increasing the expression of a rate-limiting enzyme of bile acid synthesis [23]. Taurine’s antihypertensive action is due to sympathetic modulation by attenuating signaling pathways that cause vasoconstriction and increase in blood pressure and by enhancing mechanisms that cause vasodilation [3]. The anti-inflammatory action of taurine seems to be related to the formation of taurine chloramine at the site of inflammation which regulates expression and secretion of inflammatory cytokines, inhibits nuclear factor kappa B activation, and increases adiponectin levels [24]. The suppressive effect of taurine against oxidative stress is associated with its ability to sequester reactive oxygen/nitrogen species [25], or to restore antioxidant enzymes activity and regenerate thiol group [26].

Our data suggest that the protection of taurine against stroke risk may not be appreciable in the presence of adverse effects of chronic long-term smoking. Interestingly, a recent animal study also found an interaction effect between taurine and smoking, such that taurine supplementation increased cardiac wall thickness and worsened diastolic function in rats exposed to cigarette smoke [27]. Abundant evidence demonstrates that smoking is associated with insulin resistance [28], diabetes [29], and dyslipidemia [30], all of which are well-known risk factors for stroke. It is plausible that the potential beneficial effect of taurine on stroke risk may be overshadowed by the harmful effects of smoking [31,32], the lower circulating concentrations of antioxidant micronutrients in smokers [31,32], or unhealthy diet habits among smokers [33]. This is in line with stronger effects of other nutritional factors on chronic diseases observed among never smokers [34–36]. On the other hand, a previous study demonstrated that taurine supplementation in young smokers (*n* = 15; aged 20–37 years) with a history of smoking of ≥ 2 years significantly restored impaired endothelial dysfunction [37]. Taurine may be able to attenuate early vascular damage induced by smoking and delay the development of CVD; however, the effects may be different in long-term smokers, such as those in the present study with an average of 30 years of smoking at enrollment. The capacity of taurine may be overwhelmed by an accumulation of smoking-related deleterious effects. This hypothesis, however, needs to be confirmed in future large studies.

The strengths of the present study include the prospective nature of the study design with detailed data on stroke risk factors at the individual level and the use of repeated samples for 58% of the participants to improve the reliability of taurine measurement. Serum taurine, which was positively correlated with dietary intake of poultry (r = 0.14, *p* < 0.01) [16] in our population, is likely to reflect dietary intake from foods, because blood samples were collected in 1985–86, prior to common use of energy drinks with a high level of taurine. Therefore, we cannot rule out the effects of taurine at much higher concentrations.

Several potential limitations should also be noted. Firstly, fasting blood samples may be optimal for taurine measurement. Previous research indicated that mean plasma taurine level did not differ by fasting status, although a large amount of intake may lead to a transient increase [38]. We aimed to measure usual long-term level of serum taurine. Also, although we had repeated samples for serum taurine which can increase the long-term reproducibility of serum taurine, we did not have data on changes in dietary habits or changes in serum taurine over time. The potential misclassification of taurine levels, which should not have been differential by stroke status, may have contributed to the overall null association between taurine and stroke risk. Furthermore, serum samples were stored for an average of 26 years before the analyses of taurine. However, the levels of taurine did not differ by storage time, and the association between serum taurine and stroke risk did not differ by storage time (data not shown). Secondly, our study population included 80% Caucasian women, and therefore, the study results might not be generalizable to men and other races. However, there is no evidence that the effects of taurine would differ by sex or race. The focus on women with more homogenous risk factors for stroke, however, should enhance the internal validity of the findings. Nevertheless, the range of taurine intake in one ethnic group may have been too narrow to detect an effect and our findings do not preclude that studies of various ethnic groups with widely-distributed serum taurine levels may reveal a significant protective effect of taurine on stroke risk. The ascertainment of hypertension and diabetes status was based on self-reported data, and we did not have information on blood cholesterol. The literature has indicated >80% sensitivity and specificity of these self-reported chronic conditions [39,40]. Taurine-related risk factors such as cholesterol, blood pressure, and diabetes may serve as a pathological mediator underling the effects of taurine on CVD, which should not have been controlled in the analyses. Finally, as mentioned, the number of confirmed cases of ischemic stroke and hemorrhagic stroke were limited. Future studies need to be conducted to assess the association between serum taurine and stroke risk by stroke subtypes.

In conclusion, our study does not support an association between serum taurine and stroke risk. The inverse association in never smokers needs to be confirmed in future studies.
